# Stunting and inequality in Sri Lanka compared with other low- and middle-income South Asian countries

**DOI:** 10.1017/S1368980025000205

**Published:** 2025-03-19

**Authors:** Damith Chandrasenage, William Johnson, Paula L. Griffiths

**Affiliations:** 1 School of Sport, Exercise and Health Sciences, Loughborough University, Loughborough, UK; 2 Department of Social Statistics, Faculty of Social Sciences, University of Kelaniya, Colombo, Sri Lanka

**Keywords:** Stunting, Socio-economic position, South Asia, Sri Lanka

## Abstract

**Objective:**

This study investigates and measures whether the association of childhood stunting with household socio-economic position (SEP) differs in Sri Lanka compared with other South Asian countries.

**Design:**

Secondary analysis of data of children from the latest available Demographic and Health Surveys data (survey years, 2016–2018). The exposures (SEP) were maternal education and wealth. The outcome was stunting. Binary logistic regression models incorporated SEP, country and SEP-by-country interaction terms.

**Setting:**

A nationally representative sample of children from Bangladesh, India, Nepal, Pakistan and Sri Lanka.

**Participants:**

Mothers/caregivers of children under 36 months (133 491).

**Results:**

The prevalence of stunting in Sri Lanka of 19 % was much lower than that observed for all the other low- to low–middle income South Asian countries (37 % in Bangladesh, 36 % in India, 31 % in Nepal and 30 % in Pakistan). The association of SEP with odds of stunting was similar in Sri Lanka compared with other South Asian countries. The only exception was weaker associations of wealth with stunting in Sri Lanka compared with Bangladesh. For example, in Sri Lanka, the poorest group had 2·75 (2·06, 3·67) times higher odds of stunting compared with the richest group, but in Bangladesh, this estimate was 4·20 (3·24, 5·44); the difference between these two estimates being 0·65 (0·44, 0·96) on the OR scale.

**Conclusions:**

The lower prevalence of stunting in Sri Lanka is unlikely to be due to less inequality. It is more likely that the lower prevalence of stunting in Sri Lanka is related to there being fewer mothers belonging to the lowest SEP groups.

Approximately 22 % of all children under 5 years of age were stunted in 2020 and more than one-third of these children live in South Asia^(1)^. This is important because the WHO has reported that around 45 % of deaths among children under 5 years of age are still linked to undernutrition and these mostly occur in low-and middle-income countries (WHO, 2021)^(1)^.

Stunting is associated with numerous adverse outcomes including poor child development, less productivity and chronic diseases in adulthood^(2–4)^. Existing evidence has consistently shown that better socio-economic position (SEP) is associated with lower risk of childhood stunting in South Asia^(5–7)^. This association has been demonstrated using diverse measures of SEP, such as family income, maternal education and household wealth^(3,6,8)^ including in studies from Sri Lanka^(9–11)^. SEP includes multiple levels in any society including individual, household and community levels. Stunting at the youngest ages is most likely to be affected by factors in the household environment. This is because children under 36 months spend a large amount of time at home in the South Asian context^(12)^.

Despite similar levels of economic development compared with most other South Asian countries, there is considerably less childhood stunting in Sri Lanka. For example, in Sri Lanka, the estimated prevalence of under-5 stunting was 16 % in 2020, compared against 37 % in Pakistan, 31 % in India and 30 % in both Bangladesh and Nepal^(1)^. One explanation for the lower prevalence of childhood stunting in Sri Lanka is that there is less inequality compared with that observed in other South Asian countries^(9)^. According to the UNICEF definition, the countries belonging to South Asia are Bangladesh, India, Maldives, Nepal, Pakistan, and Sri Lanka. These countries, excluding the Maldives, are low- and-middle-income countries. Although studies have investigated differences in socio-economic inequality in stunting between low- and middle-income countries, including South Asian countries^(13–18)^, none have compared inequality in stunting in Sri Lanka to that observed in other South Asian countries.

This study aims to investigate whether the association of household SEP with stunting in children under 36 months of age differs in Sri Lanka compared with other low- and middle-income South Asian countries (Bangladesh, India, Nepal and Pakistan).

## Material and methods

### Surveys

The present study used the latest available Demographic and Health Surveys (DHS) data with anthropometric data of the four South Asian countries: Bangladesh (2017/18), Nepal (2016), Pakistan (2017/18) and Sri Lanka (2016). The Indian National Family Health Survey 4 data (2015/16) with anthropometric data were used for India. The DHS and National Family Health Survey are nationally representative household surveys. The methods and types of data collected are almost identical for DHS and National Family Health Survey.

### Samples

The total number of children in the study was 133 491. The sample selection process for each country is shown in Appendix Figure 1. Mothers with multiple births were excluded from the analysis.

### Outcome

Stunting was defined as a height-for-age Z-scores according to the WHO-2006 Standards^(19)^ more than two standard deviations below the median (i.e. 50th percentile).

### Exposure

Two measures of SEP were considered; maternal education (none, primary, secondary, and higher education [referent]) and a within-country household wealth index (poorest, poorer, middle, richer, and richest [referent]). Potential confounders included in the analyses were sex of child (male or female), place of residence (urban, rural), and child’s age in months.

### Statistical analysis

Descriptive statistics were computed for each country separately. Subsequently, data from all countries were pooled together. Binary logistic regression models with stunting as an outcome were developed that incorporated SEP, country and SEP-by-country interaction terms. SEP-by-country interaction terms were included in the models to estimate how the associations of SEP with stunting differed in Sri Lanka compared with each other country. A set of interaction terms was created for mother’s education by country (e.g. no education mothers in Sri Lanka, no education mothers in Bangladesh, etc.) and another set of interaction terms was included for household wealth by country (e.g. poorest household in Sri Lanka, poorest household in Bangladesh etc.). The first set of models considered mother’s education and wealth index separately. Then a second set of models considered mother’s education and wealth index together. All models were adjusted for all potential confounders and included sampling weights. For each model, estimates were obtained showing (1) the associations (e.g. of mother’s education with stunting) in each country and (2) how the associations were different in Sri Lanka compared with each other South Asian Country.

All analyses were conducted using SPSS (Version 27).

## Results

Mean height-for-age Z-scores in Sri Lanka was –0·93 and in Bangladesh, India, Nepal and Pakistan it was between –1·5 and –1·2 (Table 1). Consequently, the prevalence of stunting in Sri Lanka of 19 % was lower than that observed for all the other low- to low–middle income South Asian countries (37 % in Bangladesh, 36 % in India, 31 % in Nepal, 30 % in Pakistan). Mothers in Sri Lanka were, on average, more educated than mothers in the other countries. For example, 54 % of mothers in Sri Lanka had higher education compared against just 11 % in India. The wealth index was calculated within country and was a relative quintile-based measure, meaning that in all countries there will be approximately 20 % of the sample in each quintile. More children in Sri Lanka (85 %) than other South Asian countries (25–73 %) lived in rural areas. More than 65 % of children in the sample in each country were over 12 months old.

**Table 1:** Descriptive statistics stratified by country^*^

	Sri Lanka *n* 4198		Bangladesh *n* 4305		India *n* 115 570		Nepal *n* 1065		Pakistan *n* 1774	
	Mean	sd	Mean	sd	Mean	sd	Mean	sd	Mean	sd
Outcome										
HAZ	−0·93	1·30	−1·50	1·39	−1·34	1·81	−1·32	1·31	−1·24	1·66
	*n*	%	*n*	%	*n*	%	*n*	%	*n*	%
Stunting										
Not stunted	3407	81·2	2699	62·7	73 451	63·6	733	68·9	1249	70·4
Stunted < –2 Z	791	18·8	1606	37·3	42 119	36·4	332	31·1	525	29·6
Exposure										
Mother’s educational										
Higher	2252	53·6	703	16·3	12 469	10·8	190	17·9	371	20·9
Secondary	1795	42·8	2058	47·8	54 481	47·1	353	33·2	436	24·6
Primary	121	2·9	1247	29·0	15 759	13·6	217	20·4	230	13·0
No education	30	0·7	297	6·9	32 861	28·4	305	28·5	737	41·5
Wealth index										
Richest	825	19·7	793	18·4	16 876	14·6	182	17·1	480	27·1
Richer	922	22·0	836	19·4	21 183	18·3	241	22·7	386	21·8
Middle	851	20·3	799	18·6	23 258	20·1	261	24·5	397	22·4
Poorer	839	20·0	898	20·9	25 566	22·1	213	20·0	291	16·4
Poorest	761	18·1	979	22·7	28 687	24·8	168	15·8	220	12·4
Covariates										
Place of residence										
Rural	3555	84·7	2893	67·2	83 977	72·7	307	28·8	442	24·9
Urban	643	15·3	1412	32·8	31 593	27·3	758	71·2	1332	75·1
Sex of child										
Male	2121	50·5	2249	52·2	59 806	51·7	592	55·6	889	50·1
Female	2077	49·5	2056	47·8	55 764	48·3	473	44·4	885	49·9
Age of child (months)										
0–5	518	12·4	702	16·3	15 073	13·0	159	14·8	301	17·0
6–11	690	16·4	668	15·5	20 012	17·3	185	17·4	282	15·9
12–23	1431	34·1	1492	34·7	40 315	34·9	379	35·7	586	33·1
24–35	1559	37·1	1443	33·5	40 170	34·8	342	32·1	605	34·1

HAZ, height for age Z-score.

* The frequencies (*n* s) in the table have been weighted using sampling weights.

Table 2 shows the estimated associations for each SEP measure considered separately. The estimated difference of the associations between the SEP measures and odds of stunting in Sri Lanka *v*. each country is shown in a separate column for each country. For example, the first column in Sril Lanka shows the mother’s secondary education group had 1·59 (95 % CI = 1·34, 1·90) times higher odds of stunting compared with the higher education group. In Bangladesh, this estimate was 2·36 (95 % CI = 1·85, 3·02). On the OR scale, the difference between these two estimates, 0·67 (95 % CI = 0·50, 0·91), is shown in the column ‘Sri Lanka *v*. Bangladesh Difference’. In all low- and low–middle income countries, the odds of child stunting were higher in (1) mothers with no education and mothers with primary education (compared with mothers with higher education) and (2) the middle, poorer and poorest categories of the wealth index (compared with the richest category). The highest association between the SEP measures and stunting in the poorest household was in Pakistan (adjusted OR = 4·61, 95 % CI = 2·81, 7·55). The lowest association between the SEP measures and stunting was in the richer household in India (adjusted OR = 1·42, 95 % CI = 1·31, 1·54). The associations of the SEP measures with the odds of stunting were similar in Sri Lanka compared with all other South Asian countries, except Bangladesh. The estimates for the wealth index were consistently lower in Sri Lanka than in Bangladesh. For example, in Sri Lanka, the poorest group had 2·75 (95 % CI = 2·06, 3·67) times higher odds of stunting compared with the richest group, but in Bangladesh, this estimate was 4·20 (95 % CI = 3·24, 5·44). On the OR scale, the difference between these two estimates of 0·65 (95 % CI = 0·44, 0·96) provides evidence of a weaker association in Sri Lanka between wealth index and stunting compared with Bangladesh. This association was consistent in each wealth index group in Sri Lanka compared with Bangladesh. The association of maternal education with higher odds of stunting was also lower in Sri Lanka than in Bangladesh but only at secondary (*v*. higher) level. The estimated OR difference was 0·67 (95 % CI = 0·50, 0·91).

**Table 2:** Associations of household socioeconomic position variables (considered separately) with the odds of stunting for each country, with estimated differences (in the associations) between Sri Lanka and each other country

	Sri Lanka		Bangladesh		Sri Lanka *v*. Bangladesh Difference		India		Sri Lanka *v*. India Difference		Nepal		Sri Lanka *v*. Nepal Difference		Pakistan		Sri Lanka *v*. Pakistan Difference	
	AOR	95 % CI	AOR	95 % CI	AOR	95 % CI	AOR	95 % CI	AOR	95 % CI	AOR	95 % CI	AOR	95 % CI	AOR	95 % CI	AOR	95 % CI
Model 1																		
Mothers education																		
Higher [referent]	–		–		–		–		–		–		–		–		–	
Secondary	1·59	1·34, 1·90	2·36	1·85, 3·02	0·67	0·50, 0·91	1·67	1·55, 1·80	0·95	0·79, 1·16	1·39	0·86, 2·25	1·15	0·69, 1·91	1·88	1·09, 3·22	0·85	0·48, 1·50
Primary	2·74	1·81, 4·14	2·79	2·15, 3·61	0·98	0·60, 1·60	2·45	2·25, 2·66	1·12	0·73, 1·70	2·23	1·33, 3·75	1·23	0·63, 2·38	2·22	1·24, 3·98	1·23	0·60, 2·52
No education	4·20	1·92, 9·20	3·65	2·58, 5·16	1·15	0·49, 2·71	3·18	2·95, 3·42	1·32	0·60, 2·91	2·55	1·59, 4·09	1·65	0·66, 4·12	3·93	2·43, 6·34	1·07	0·43, 2·68
Model 2																		
Wealth Index																		
Richest [referent]	–		–		–		–		–		–		–		–		–	
Richer	1·32	0·96, 1·80	2·14	1·63, 2·81	0·62	0·41, 0·93	1·42	1·31, 1·54	0·93	0·67, 1·28	1·77	0·97, 3·24	0·74	0·38, 1·46	1·29	0·79, 2·11	1·02	0·57, 1·81
Middle	1·69	1·25, 2·30	3·05	2·32, 4·00	0·56	0·37, 0·84	1·97	1·83, 2·13	0·86	0·63, 1·17	2·28	1·26, 4·13	0·74	0·38, 1·45	1·99	1·27, 3·13	0·85	0·49, 1·47
Poorer	1·96	1·46, 2·64	4·25	3·27, 5·51	0·46	0·31, 0·68	2·67	2·48, 2·88	0·74	0·54, 1·00	2·71	1·49, 4·92	0·73	0·37, 1·41	2·83	1·79, 4·49	0·69	0·40, 1·20
Poorest	2·75	2·06, 3·67	4·20	3·24, 5·44	0·65	0·44, 0·96	3·55	3·30, 3·82	0·77	0·58, 1·04	4·58	2·49, 8·40	0·60	0·31, 1·18	4·61	2·81, 7·55	0·60	0·34, 1·05

AOR, adjusted OR.

All models included sampling weights and were adjusted for age, sex (binary term: male and female) and place of residence (binary term: urban and rural). Age was two linear spline terms as children under 22 months old tended to more stunting than older (22–35 months) children.

The estimated differences in the odds scale have been calculated using SEP-by-country interaction terms included in each regression model. Regression models were estimated using STATA software. See the statistical analysis sub-section in the Material and Methods for more details.

Table 3 shows the estimated differences between Sri Lanka and each of the other South Asian countries combined into one model to test the association between each of the SEP measures (mother’s education and wealth) and the odds of stunting. Results show similar but slightly weaker estimates for the associations of wealth and mother’s education with stunting compared with Table 2. Sri Lanka still had a weaker association of wealth (middle to poorest level) with odds of stunting compared with Bangladesh (e.g. Sri Lanka *v*. Bangladesh difference OR in the poorest level was 0·64 (95 % CI = 0·42, 0·97).

**Table 3:** Associations of household socio-economic position variables (considered together) with the odds of stunting for each country, with estimated differences (in the associations) between Sri Lanka and each other country

	Sri Lanka		Bangladesh		Sri Lanka *v*. Bangladesh difference		India		Sri Lanka *v*. India difference		Nepal		Sri Lanka *v*. Nepal difference		Pakistan		Sri Lanka *v*. Pakistan difference	
	AOR	95 % CI	AOR	95 % CI	AOR	95 % CI	AOR	95 % CI	AOR	95 % CI	AOR	95 % CI	AOR	95 % CI	AOR	95 % CI	AOR	95 % CI
Mothers Education																		
Higher [referent]	–		–		–		–		–		–		–		–		–	
Secondary	1·32	1·09, 1·61	1·67	1·29, 2·17	0·79	0·57, 1·10	1·36	1·26, 1·47	0·97	0·79, 1·20	1·17	0·71, 1·94	1·13	0·66, 1·93	1·75	1·00, 3·05	0·76	0·42, 1·37
Primary	1·97	1·28, 3·03	1·66	1·24, 2·20	1·19	0·71, 1·99	2·02	1·86, 2·20	1·16	0·74, 1·80	1·84	1·10, 3·08	1·16	0·56, 2·40	2·73	1·59, 4·69	1·03	0·49, 2·17
No education	2·88	1·26, 6·55	2·03	1·39, 2·96	1·42	0·57, 3·50	1·70	1·55, 1·86	1·42	0·62, 3·26	1·69	0·94, 3·04	1·56	0·59, 4·14	1·90	1·04, 3·47	1·05	0·39, 2·82
Wealth index																		
Richest [referent]	–		–		–		–		–		–		–		–		–	
Richer	1·22	0·89, 1·67	1·91	1·44, 2·52	0·69	0·45, 1·06	1·25	1·15, 1·36	1·01	0·73, 1·40	1·47	0·78, 2·77	0·65	0·31, 1·38	1·06	0·64, 1·75	0·89	0·47, 1·69
Middle	1·51	1·10, 2·08	2·56	1·93, 3·40	0·51	0·33, 0·77	1·54	1·42, 1·68	0·91	0·65, 1·26	1·65	0·86, 3·18	0·80	0·39, 1·64	1·42	0·88, 2·31	0·98	0·54, 1·78
Poorer	1·68	1·23, 2·31	3·33	2·52, 4·40	0·59	0·39, 0·90	1·86	1·70, 2·03	0·98	0·71, 1·36	2·10	1·10, 4·01	0·91	0·44, 1·89	1·71	1·03, 2·83	1·06	0·60, 1·90
Poorest	2·17	1·58, 2·99	3·13	2·36, 4·16	0·64	0·42, 0·97	2·15	1·96, 2·36	0·97	0·70, 1·35	3·34	1·70, 6·60	0·83	0·41, 1·67	2·45	1·40, 4·28	1·15	0·63, 2·08

AOR, adjusted OR.

All models included sampling weights and were adjusted for age sex (binary term: male and female) and place of residence (binary term: urban and rural).

## Discussion

The key finding of this study is that the associations of mother’s education and wealth with odds of stunting were generally similar in Sri Lanka compared with other South Asian countries. This suggests that the observed lower levels of child stunting in Sri Lanka compared with other low- and middle-income countries (LMIC) in South Asia are not likely because of less inequality in stunting. The exception was the weaker association of the wealth index with odds of stunting in Sri Lanka compared with Bangladesh.

The reason for finding weaker associations of wealth index with odds of stunting in Sri Lanka compared with Bangladesh may be due to households in the poorest wealth group in Sri Lanka being less poor than the households in the same wealth group in Bangladesh. The asset-based DHS wealth index is similar for Sri Lanka and Bangladesh, in terms of composition of the variables (https://dhsprogram.com), but it is a relative index within country and it is likely that the lowest level in the index indicates greater poverty in Bangladesh than Sri Lanka. Globally, socio-economic indicators show less poverty, but higher variability in wealth in Sri Lanka compared with Bangladesh. For example, extreme poverty (the population below the international poverty line, $1·90 per day per person) in Sri Lanka was only 0·8 % in 2016 and it was 15 % in Bangladesh. However, inequality was higher in Sri Lanka with a reported higher Gini index (higher value indicates higher inequality) of 39·3 compared with 32·4 in Bangladesh^(20)^. This information shows that greater inequality could be in Sri Lanka although our study revealed a relatively lower prevalence at the national level of Sri Lanka compared with other low- and middle-income countries. For example, we found that the odds of child stunting in Sri Lanka are higher in children with no education mothers and also in children living in the poorest households compared with the highest SEP groups. This evidence has also been confirmed by a subpopulation study in the eastern province of Sri Lanka (Sujendran et al., 2015)^(21)^ reporting a higher inequality in the lower SEP groups (wealth and mother’s education) than in the highest SEP groups. For example, Sujendran et al. reported a nearly four times higher risk of stunting in children if their parent’s education level is below the secondary level compared with the above secondary level (OR = 4·91, *P* = 0·048).

Our results suggest that the observed lower levels of stunting in Sri Lanka are likely to be due to fewer children/mothers in the most deprived groups. For example, less mothers in Sri Lanka had no education or primary education compared with the other low- and low–middle-income South Asian countries. Higher education is likely associated with a mother’s ability to follow the directions of health care practitioners and to support and care for their children to promote optimal early growth and development. The relationship between education and better health and nutrition practices concurs with other local literature which reports that educated mothers in Sri Lanka are more likely to follow nutrition related instructions given by health caregivers and in turn have lower levels of stunting (e.g. Jayawardena 2012)^(11)^. Sri Lanka reports better child feeding indicators compared with other South Asian countries. For example, the regional highest exclusive breast-feeding rate (90·3 %) and highest solid or semisolid complementary foods feeding rate (97·5 %) in children aged 6–23 months was in Sri Lanka^(22)^. These indicators for other South Asian countries ranged, respectively, from 19·6 % (Pakistan) to 59·8 % (Bangladesh), and 74·5 % (India) to 95·9 % (Nepal),^(23–26)^.

Gender equality in education and positive attitudes and cultural and social norms about girls’ education have given more education opportunities for women in Sri Lanka compared with other South Asian countries. A formal education system with education opportunities for girls in Sri Lanka was initiated earlier in Sri Lanka than other South Asian countries. For example, Sri Lankan children have had opportunities for education irrespective of sex, race, religion, caste or class since the colonial periods of the Portuguese (1505–1658), Dutch (1658–1796), and British (1796–1948)^(27)^. In contrast, there has been less equality of education by gender across the other South Asia countries^(27,28)^. A free education policy was introduced in Sri Lanka earlier (in the year 1945) than for the other South Asian countries (1990 in Bangladesh, 2009 in India, 2007 in Nepal and 2010 in Pakistan),^(29,30)^. This is likely to have further enhanced women’s education opportunities in Sri Lanka compared with other South Asian countries. These benefits are likely magnified for current mothers in Sri Lanka with transfer of education benefits over generations.

Findings that Sri Lanka observes the same level of inequality in stunting compared with other low- and low–middle income countries in South Asia in the analysis means that there remain vulnerable groups in Sri Lanka that have a risk of stunting. For example, stunting in the estate worker community in Sri Lanka (32 %) is higher than other rural (17 %) and urban (15 %) areas according to the SLDHS-2016^(22,31)^. Furthermore, the current political and economic crisis in Sri Lanka will create more challenges for preventing stunting because the crisis has the potential to move larger proportions of the population into poverty. This could break down the benefits of education and health that have been achieved to reduce child stunting in Sri Lanka.

Interventions to improve wealth and mother’s education are likely to reduce stunting in all South Asian countries. For example, Mishra *et al.*, (2019)^(32)^ showed that scaling up existing household wealth levels to the level of the richest quantile in the population had the potential to reduce under-5 stunting in Odisha state in India by 4 % from 2015 to 2030. An overview paper of Torlesse & Aguayo (2018)^(33)^ summarised the evidence of the implications for the direction of future advocacy, policy and program actions to improve maternal and child nutrition in South Asia. They reported a need for intervention to reinforce girls’ access to education in contexts where they are deprived of educational opportunities in South Asia. However, evidence shows that there is a lack of intervention programs to improve the coverage of maternal nutrition interventions in South Asia^(34)^. Identifying policy differences in education between South Asian countries is, therefore, needed to facilitate creating environments more conducive to optimal child growth and development in other South Asian countries.

The strengths of this research are the use of the large samples consistently collected using DHS measures across these South Asian countries to facilitate a coherent comparison. A limitation of these data is that it is not possible to look at causal relationships because the surveys are cross sectional. The analysis was also constrained to the indicators collected in the surveys such as the use of a wealth index and measures of education that are collected in DHS surveys. The number of children with mothers in the ‘no education’ group in Sri Lanka is small and the 95 % CI are wide. There are some limitations in using asset-based wealth indices across countries because the indices are relative within country and the value of an asset and its importance may vary widely across country/geographical area and time^(35,36)^. The study was therefore not able to formally estimate the extent to which the lower rates of stunting in Sri Lanka are due to less inequality.

### Conclusion

Our results suggest that the lower prevalence of child stunting in Sri Lanka compared with other low- and middle-income countries in South Asia is unlikely due to less inequality (i.e. weaker associations of maternal education and household wealth with the odds of stunting). Instead, the lower prevalence of child stunting in Sri Lanka is likely due to fewer children/mothers belonging to the most deprived SEP groups.

## Supporting information

Chandrasenage et al. supplementary material Chandrasenage et al. supplementary material

## Supplementary material

For supplementary material/s referred to in this article, please visit https://doi.org/10.1017/S1368980025000205
