# Supplementary material for: Stunting and inequality in Sri Lanka compared with other low- and middle-income South Asian countries
Source: Public Health Nutr. 2025 Mar 19;28(1):e63. doi: 10.1017/S1368980025000205 (PMC12086723; doi:10.1017/S1368980025000205)
Supplement: Chandrasenage et al. supplementary material [file S1368980025000205sup001.docx]

**Figure Legend (Supplementary)**

BD= Bangladesh, IN=India, NP= Nepal, PK= Pakistan, SL= Sri Lanka, All= Total for all South Asian countries.

*Height out of plausible limits; reported height is out of plausible limits of height of a child, and Age in days out of plausible limits; the given age is out of possible limits by dates (decided by a DHS data analytical team after confirming by carefully checking the related data files together such as women (IR) file, household (HR) file and person (PR) file). Children with height-for-age-z-score more than or less than six (+6 SD <HAZ<-6SD) standard deviations were removed from the sample as extreme outliers.

†Height missing (Not measured); Height is not measured at the survey as dead, sick, not present, refused, mother refused, no measurement found in household for other reason.

Child lives with whom, respondent’s current age, respondent currently working were later excluded from the data set. More than 99% of the persons living with child was mother being nearly a constant variable, maternal age is highly correlated with birth order includes in the analysis.
